# Mechanical extraction of chronic venous thrombus using a novel device: a report of two cases

**DOI:** 10.1016/j.jvscit.2022.09.011

**Published:** 2022-09-29

**Authors:** Raja Ramaswamy, Akshay Guttikonda, Michael D. Kaplan

**Affiliations:** a Community Health Network, Community North and East Hospitals, Indianapolis, IN; b Inova Alexandria Hospital, Alexandria, VA

**Keywords:** Chronic thrombus, Deep vein thrombosis, Endovascular intervention, Mechanical thrombectomy

## Abstract

In deep vein thrombosis (DVT), the structure and composition of the venous thrombus can change rapidly over time. Studies have shown mixed results with anticoagulant and thrombolytic therapies, and the issue will be exacerbated in the case of chronic DVT (defined as thrombus still present after ≥4 weeks of failed treatment after a DVT diagnosis), with no well-accepted interventions. In the present report, we have described two patients in whom mechanical thrombectomy with a novel device was used to remove extensive, chronic thrombus. At follow-up, both patients showed improved blood flow and patency with resolution of their edema and pain. Because thrombus can often be more chronic than expected from a patient’s medical history alone, mechanical intervention as the first approach might be warranted.

The thrombus composition in patients with deep vein thrombosis (DVT) is everchanging. Within 3 weeks of the initial formation, the thrombus could be composed of ≤80% collagen.^1^ As this structural transformation progresses, thrombolytic efficacy will deteriorate because thrombolytic drugs cannot dissolve or break up chronic, collagenous material. Consequently, three major clinical studies of patients with DVT had constrained inclusion criteria that limited patients to those with symptoms for <14 days (ATTRACT [acute venous thrombosis: thrombus removal with adjunctive catheter-directed thrombolysis],^2^ CAVA [ultrasound-accelerated catheter-directed thrombolysis on preventing post-thrombotic syndrome]^3^) or <21 days (CaVenT [catheter-directed venous thrombolysis in acute iliofemoral vein thrombosis]^4^). Thus, the real-world applicability of these studies has also been limited. However, a mechanical device that does not require adjunctive thrombolytic agents would bypass these limitations.

The ClotTriever System (Inari Medical, Irvine, CA) is a mechanical thrombectomy device and has been shown to be effective in successfully removing thrombus from acute, subacute, and chronic lesions.^5^^,^^6^ A comprehensive review of the literature revealed a limited but growing body of reported studies on the use of mechanical thrombectomy to treat chronic DVT. Dexter et al^5^ reported that mechanical thrombectomy resulted in positive thrombus removal, procedural outcomes, and safety outcomes at 30 days that were sustained in the long-term outcomes at 6 months. Maldonado et al^6^ showed that although 49.0% of extracted thrombus was more chronic than the patient symptom duration had suggested, mechanical thrombectomy was effective in removing thrombus of all chronicities. Mouawad^7^ reported the case of a patient with chronic thrombus who had experienced complete resolution of decades-long ulceration and avoided amputation after successful treatment with mechanical thrombectomy, demonstrating the promise of this procedure.

The ClotTriever BOLD catheter (Inari Medical) is an addition to the system, with a more robust coring element designed for more intractable and chronic thrombus. The device has a stronger radial force, is slightly more aggressive, and could be one of the tools used to treat chronic thrombus. To the best of our knowledge, the present report is the first of this novel device. We have reported the outcomes of two patients with chronic thrombus in whom anticoagulation therapy or thrombolytic agents, or both, had failed. We have also elaborated on the underlying pathophysiology to assist physician decision-making. The patients provided written informed consent for the report of their case details and imaging studies.

## Case report

### Patient 1

A 64-year-old man had presented with persistent right leg edema with pain 6 weeks after a diagnosis of extensive right lower extremity DVT. Duplex ultrasound imaging at that time had demonstrated a completely occluded distal right external iliac vein (EIV), common femoral vein (CFV), proximal profunda, above-the-knee greater saphenous vein, popliteal vein, paired peroneal veins, and paired posterior tibial veins. The patient had a history of portal vein thrombosis, left-sided pulmonary embolism, and thrombocytopenia for which he had been prescribed warfarin and, subsequently, low-molecular-weight heparin but had become noncompliant. A decision was made to perform ultrasound-assisted catheter-directed thrombolysis (EKOS Endovascular System; Boston Scientific, Marlborough, MA) to first establish distal to proximal inflow and support more proximal patency.

**Fig 1:**
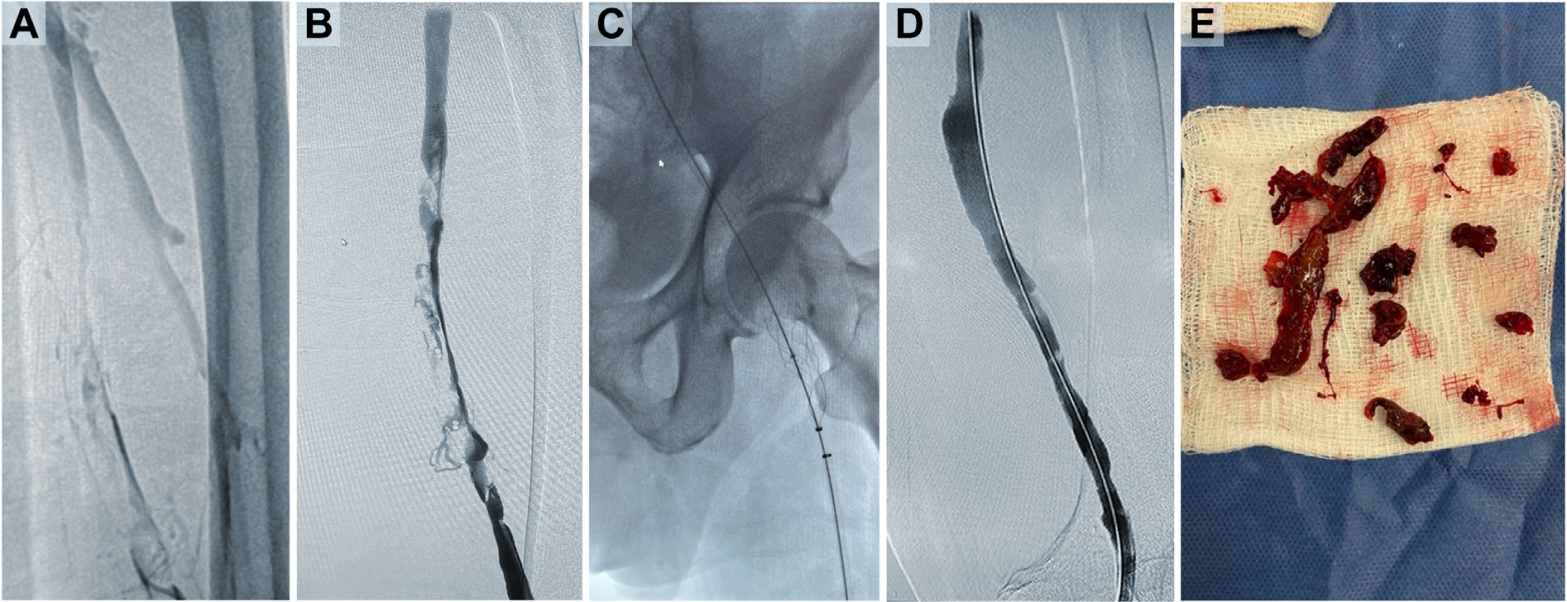
**A,** Venogram before lysis demonstrating chronic thrombus in the femoral vein and adjacent filling of the profunda femoral vein. **B,** After lysis, residual chronic thrombus can be seen throughout the right femoral with no filling of the profunda femoral vein. **C,** ClotTriever BOLD catheter deployed in the inferior vena cava (IVC) before retraction. **D,** Repeat venogram showing a widely patent femoropopliteal segment. **E,** Chronic thrombus extracted from the patient.

One of the paired right posterior tibial veins was accessed and venography performed, revealing that the popliteal vein was completely occluded with thrombus proximally. The femoral vein was occluded distally, and the right EIV was patent with nonocclusive, wall-adherent chronic thrombus (Fig 1, *A*). Overnight lysis was initiated.

The EKOS catheter was removed the next day, and venography showed improvement but with persistent areas of narrowing and chronic thrombus. The right femoropopliteal segment had residual chronic thrombus throughout, with flow-limiting synechiae, webbing, and multiple areas of tight narrowing (Fig 1, *B*).

Given the extensive chronic thrombus, we began mechanical thrombectomy with the ClotTriever System. The right popliteal vein was accessed under ultrasound guidance in accordance with the manufacturer’s instructions for use. A 13F ClotTriever sheath was placed into the right popliteal vein and advanced over a guidewire with the distal tip superior to the inferior vena cava (IVC) confluence. A ClotTriever BOLD catheter was advanced through the sheath, deployed in the IVC, and then retracted (Fig 1, *C*), collecting and extracting dense, chronic thrombus in five passes (Fig 1, *E*).

Repeat venography demonstrated significant improvement in the right femoropopliteal segment with some mild residual narrowing. After 10-mm balloon dilatation, the segment was widely patent with antegrade flow and no significant residual collateral flow (Fig 1, *D*). The right CFV, EIV, common iliac vein (CIV), and IVC were all patent. Given this clearance, intravascular ultrasound was not deemed necessary. The total device time was 20 minutes, and the total procedure time was 60 minutes. Visual inspection via venography confirmed that all thrombus had been removed. The patient was discharged on the same day. Two days after the procedure, he reported symptom improvement, and at the 8-week follow-up, no DVT was seen on ultrasound and the patient reported complete resolution of his symptoms.

### Patient 2

A 51-year-old man had presented with COVID-19 (coronavirus disease 2019) pneumonia, complicated by right retroperitoneal hematoma due to anticoagulation therapy. He had developed a subsegmental pulmonary embolism, and an IVC filter had been placed. He had presented to the emergency department 7 weeks later with fever and worsening abdominal and back pain. The abdominal computed tomography (CT) findings were concerning for superinfection, and the fluid collection was subsequently drained by the interventional radiologist. The patient was discharged with a 1-month prescription for enoxaparin that was then transitioned to rivaroxaban.

**Fig 2:**
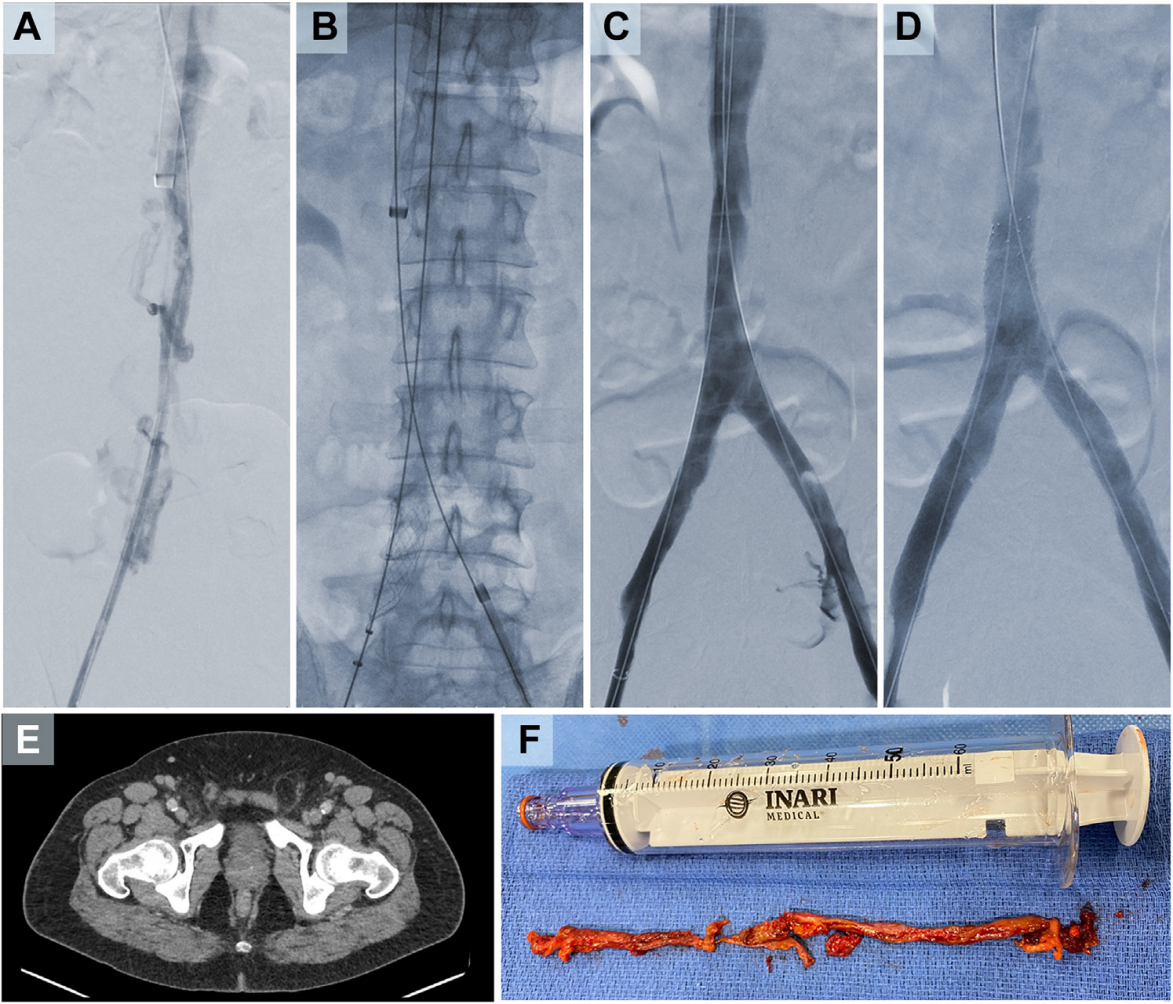
**A,** Venogram demonstrating chronic occlusion below the inferior vena cava (IVC) filter. **B,** After serial dilation and sheath placement, a ClotTriever BOLD catheter was advanced beyond the thrombus and expanded into the vessel. **C,** Venogram after thrombectomy confirming restored patency of the IVC. **D,** Digital subtraction angiography showing restored patency and flow to the bilateral common iliac veins (CIVs) and IVC. **E,** Computed tomography (CT) scan performed after the procedure demonstrating patent bilateral common femoral veins (CFVs). **F,** Chronic-appearing thrombus extracted from the patient.

The patient presented for IVC filter removal 7 weeks after discharge and was found to have bilateral lower extremity swelling. An initial venogram demonstrated chronic occlusion extending from the bilateral CFVs to the bilateral CIVs and to below the IVC filter (Fig 2, *A*), prompting a decision to reschedule the procedure to allow planning for thrombectomy with the ClotTriever system. The patient returned 3 weeks later for outpatient IVC filter removal, recanalization of the iliocaval segments via mechanical thrombectomy, and possible placement of a Food and Drug Administration-approved stent in the chronically occluded segments.

The patient was placed supine, and the right internal jugular vein was accessed under ultrasound guidance. A CT venogram was obtained before the procedure to evaluate the location and extent of the thrombus. Because the thrombus was chronic and the risk of embolization was deemed low, we decided to remove the IVC filter first using endobronchial forceps advanced via a 16F sheath. The venogram performed after IVC filter removal demonstrated no damage to the IVC.

Recanalization was performed from the bilateral CFV access owing to its proximity to the location of the thrombosis and to ensure increased support when crossing highly organized thrombus. Access to the right CFV was achieved using ultrasound guidance with a 19-gauge needle. A guidewire (Glidewire Advantage; Terumo Interventional Systems, Tokyo, Japan) was advanced within the CFV. The tract was dilated, and a 10F sheath was advanced into the proximal CFV. Recanalization was performed using a 5F catheter and guidewire. The same steps were repeated to access and recanalize the left CFV (Fig 2, *B*).

A 14-mm balloon was used to perform angioplasty of the IVC, with a 12-mm balloon used for the right and left CIVs, and a 10-mm balloon used for the CFV. After serial dilation, a 16F ClotTriever sheath was advanced into the right CFV and a 13F ClotTriever sheath into the left CFV. A ClotTriever BOLD catheter was advanced beyond the thrombus, expanded into the vessel, and retracted. Four passes were made on each side, yielding significant amounts of chronic-appearing thrombus (Fig 2, *F*). Venography performed after thrombectomy demonstrated restored IVC patency (Fig 2, *C*). Intravascular ultrasound confirmed that the infrarenal IVC was free of thrombus. Two 14-mm × 140-cm stents (Zilver Vena; Cook Medical Inc, Bloomington, IN) were placed into the bilateral CIVs. Venoplasty was performed before and after placement.

Final digital subtraction angiography demonstrated restored patency and flow into the bilateral CIVs and IVC (Fig 2, *D*). After the procedure, a CT scan demonstrated patent bilateral CFVs (Fig 2, *E*). All catheters and wires were removed. Hemostasis was achieved by manual compression. The total procedure time was ∼3 hours, and the total device time was ∼45 minutes. The patient was discharged the same day.

At 8 days after the procedure, his lower extremity swelling had improved considerably, and the pain had resolved. At 8 weeks after the procedure, CT scan confirmed patent iliac stents, without stenosis or residual thrombus. No thrombus was present in the IVC. The patient was doing well at the 3- and 6-month clinical follow-up.

## Discussion

Increasing evidence has suggested that the extracted thrombus will often be older and more chronic than the DVT symptoms would suggest.^6^^,^^7^ The outcomes from the ClotTriever Outcomes registry have shown that ∼50% of patients will have thrombus that is more chronic than that determined by the symptom assessment alone.^6^

In the present case series, the initial course of treatment—catheter-directed thrombolysis for patient 1 and anticoagulation therapy for patient 2—had done little to dissolve the thrombus or prevent it from evolving into more collagenous material. The structural transformation had progressed beyond the ability of these treatments to have any meaningful effects on the acute or long-term outcomes. A recent study reported that despite patients having an acute onset of symptoms, 91.7% of the thrombi were classified as “old” or chronic via magnetic resonance venography. As a treatment of DVT, catheter-directed thrombolysis was found to have failed a significant proportion of the time, with a success rate of only 57.1%, which had decreased precipitously to 16.7% for old, or chronic, DVT.^8^ Additionally, major bleeding had occurred in 5.4% of the patients.^8^

For our two patients, a mechanical thrombectomy solution was able to extract nearly all of the thrombus, regardless of its age or chronicity. The device selected was designed for improved thrombus engagement, with a greater radial force and better wall apposition than its predecessor. For patient 1, extensive chronic material had been extracted after multiple passes with the ClotTriever BOLD catheter, completely restoring flow through the previously occluded femoral–popliteal segment. For patient 2, complete clearance of the IVC allowed the operator to forgo the need to stent those segments. These results have shown that mechanical thrombectomy with the ClotTriever BOLD catheter could be a viable solution to treat chronic DVT proximal to the popliteal vein.

## Conclusions

Our results have shown that complete mechanical extraction of chronic thrombus with the ClotTriever System can be achieved in patients for whom anticoagulation therapy or thrombolytic agents, or both, had failed. Given the positive outcomes achieved, we propose that the ClotTriever system should be considered as a first-line approach to intervention for patients with chronic or persistent DVT.
